# Data acquisition and imaging using wavelet transform: a new path for high speed transient force microscopy†

**DOI:** 10.1039/d0na00531b

**Published:** 2020-09-10

**Authors:** Amir Farokh Payam, Pardis Biglarbeigi, Alessio Morelli, Patrick Lemoine, James McLaughlin, Dewar Finlay

**Affiliations:** Nanotechnology and Integrated Bioengineering Centre (NIBEC), School of Engineering, Ulster University Jordanstown Shore Road Northern Ireland BT37 0QB UK a.farokh-payam@ulster.ac.uk

## Abstract

The unique ability of Atomic Force Microscopy (AFM) to image, manipulate and characterize materials at the nanoscale has made it a remarkable tool in nanotechnology. In dynamic AFM, acquisition and processing of the photodetector signal originating from probe–sample interaction is a critical step in data analysis and measurements. However, details of such interaction including its nonlinearity and dynamics of the sample surface are limited due to the ultimately bounded bandwidth and limited time scales of data processing electronics of standard AFM. Similarly, transient details of the AFM probe's cantilever signal are lost due to averaging of data by techniques which correlate the frequency spectrum of the captured data with a temporally invariant physical system. Here, we introduce a fundamentally new approach for dynamic AFM data acquisition and imaging based on applying the wavelet transform on the data stream from the photodetector. This approach provides the opportunity for exploration of the transient response of the cantilever, analysis and imaging of the dynamics of amplitude and phase of the signals captured from the photodetector. Furthermore, it can be used for the control of AFM which would yield increased imaging speed. Hence the proposed method opens a pathway for high-speed transient force microscopy.

## Abstract 2

This paper introduces a fundamentally new approach for dynamic AFM data acquisition and imaging based on applying the wavelet transform on the data stream from the photodetector.

## Graphical abstract

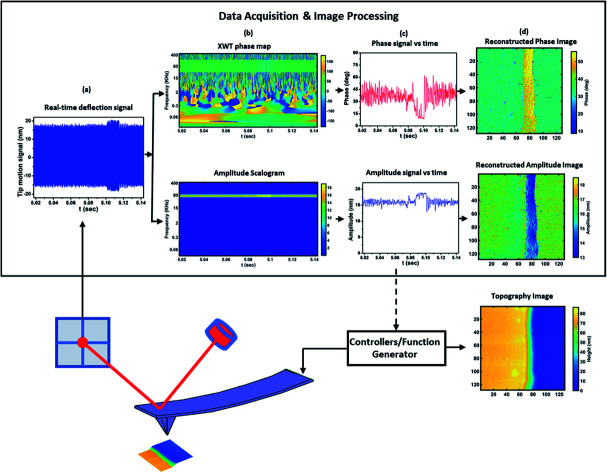

## 1. Introduction

Atomic force microscopy is a versatile tool for imaging and characterization of the electrical, mechanical, physical and chemical properties of materials at the nanometer scale with atomic resolution.^1–9^ The AFM working principle is based on the interaction between the cantilever probe system and the surface, and particularly in amplitude modulation AFM – one of the most used AFM modes – such interaction is sensed by vibrating the probe's cantilever near resonance. Vibration information, especially the amplitude which is used by the feedback system to follow the surface, is embedded in the cantilever-tip motion signal that is detected by a photodiode detector. The data stream from the photodetector is processed *via* a data acquisition and processing system that explores the information on local properties and structure.^3,9–12^ The exceptional capability of AFM to image, characterize and manipulate materials in different environments including vacuum, air and liquid with a remarkable signal-to-noise ratio triggered the development of advanced AFM techniques. In the three decades after its invention, much attention has been focused on the development of low noise platforms, high quality and fast cantilever probes, improvement of nano-positioning speed, measurement precision and bandwidth.^13–27^ Despite remarkable improvements in the increasing speed of AFM which has led to the development of high-speed AFM,^28^ relatively little effort has been devoted to improvements in information transfer, high-speed amplitude detection and transient dynamics of the cantilever during interaction with the sample under evaluation. The conventional method for amplitude calculation during AFM operation relies on the use of a lock-in amplifier (LIA),^29–31^ which is not suitable for high-speed operation. The main limitation of a LIA in calculation of amplitude for high-speed operation is the low-pass filter which not only limits the bandwidth but also results in loss of information in transient responses. In order to further improve the measurement speed, alternatives to using a LIA such as the peak-hold method,^32^ Fourier analysis,^33^ real-time integration,^34,35^ Lyapunov estimation^36^ and Kalman filter approach^37^ have been proposed. However, although these methods can speed up the process of amplitude estimation, the dynamic information on cantilever interaction is strictly limited to only several measured parameters. Although for linear systems this mechanism is acceptable, complex multidimensional cases – such as non-linearities, multifrequency, couplings of modes and transient response – cannot be solved thoroughly. All these cases carry information about the material's properties that cannot be skipped or effectively squeezed.^10^

Recording the amplitude and phase response of the cantilever with respect to the real time for different harmonics and frequencies in both transient and steady state regimes can lead to an increase of the speed and precision of feedback control. Together, with development of advanced multifrequency techniques, this would result in the possibility of extracting more detailed information from the tip–sample interaction – especially in cases where the dynamic response of the sample is relevant – and exploring sample properties and structures during imaging in more detail. This opens the possibility for transient force microscopy which would record the information conveyed by the sensing tip as a transient motion of the cantilever, in contrast to steady state operation used in standard dynamic techniques.^38–44^ In the steady state analysis, in addition to the display of the images based on the averaging of the data per pixel, spectral analysis provided by standard techniques, such as Fourier transform (FT), provides an averaged spectrum which is integrated over the whole acquisition time.^41–44^ So, in order to analyze the whole dynamics of the non-stationary signals from the photodetector, an approach that is capable of combining time domain and frequency domain analysis is mandatory. The wavelet transform (WT) method overcomes the imposed constraints by using the wavelet as the basis function.^45^ Using the WT, not only can the steady state response of the cantilever be detected, but also the whole dynamics – including transient and higher frequency responses – are captured and recorded. Previously, the WT has been used in AFM for the analysis of scanned images,^46^ eigenmodes and energy dissipation for force spectroscopy,^43,44,47–52^ characterization of passive microrheology of living myoblasts^53^ and investigation of the amplitude of higher harmonics in tapping mode AFM.^41,42^

In this paper, for the first time, wavelet theory is used to analyze the multidimensional dynamics of both amplitude and phase of the cantilever signal acquired during imaging. The captured data are used to explore the probe–sample interaction and use the transient dynamics to provide amplitude and phase images of the sample. Working on simulated data, we demonstrate that this method is effective in detecting the transient response in relation to variation in sample properties under different environmental conditions. Finally, by applying the WT on the data stream from the AFM photodetector, the resulting amplitude and phase images are shown to be in agreement with – and indeed surpassing in resolution – the ones obtained by lock-in techniques embedded in the AFM system used for the experiments.

This could be a breakthrough in the development of AFM technology towards detection of the whole dynamic response of amplitude and phase of the cantilever. It would give the ability to capture images in both transient and steady state regimes and provide the opportunity for full acquisition of cantilever data during experiments, while in current lock-in amplifier based AFM systems only the steady state values of amplitude and phase are extracted and averaged to provide the corresponding images and control the AFM. The real-time implementation of the proposed technique can significantly improve the speed of AFM operation and capability of multifrequency methods. Using the detected real-time amplitude in the imaging and control of AFM, which requires the use of application specific hardware (*e.g.* FPGA^54^) to achieve the levels of performance necessary, can lead to the new concept of high-speed transient force microscopy in AFM technology.

## 2. Wavelet transform method

The wavelet transform (WT) can transform a time domain signal into a representation that can illustrate the signal information more concisely whilst also highlighting information that was not apparent in the original signal. The WT is defined as the convolution of a signal, *x*(*t*), and a localized wavelike function, *Ψ*(*t*), also known as the mother wavelet. Mother wavelets have finite energy and should satisfy the admissibility condition, which states that the wavelet has no zero-frequency component, and therefore the mother wavelet has a zero mean. The WT is then achieved by local matching of the translated and dilated mother wavelet with the signal.^55^

The Continuous Wavelet Transform (CWT) is the sliding convolution of a signal, *x*(*t*), and the mother wavelet, *Ψ*(*t*), defined as follows (eqn (1)):1
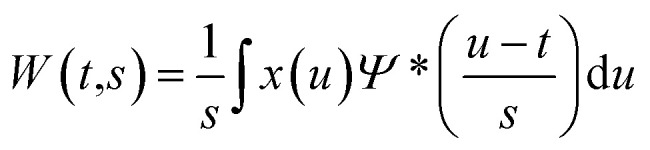
where *s* and *t* are the scale and time shift of the mother wavelet, *Ψ*, respectively and Ψ* denotes the complex conjugate of *Ψ*. The daughter wavelet, *W*(*t*,*s*), is the wavelet coefficient of the signal localized in (*t*,*s*). Therefore, the CWT presents a time–scale analysis which characterizes the power of different scales against time. The plot that is generated using the magnitude of daughter wavelet coefficients represents the spectral energy of the signal and is called a scalogram.^56^

The CWT uses scales as an alternative to frequency and decomposes a signal into a time–scale plane and each scale contains a range of frequencies. The transform of the signal into each scale is performed by a wavelet band-pass filter localized in frequency, *ω*_s_. The relative frequency, Δ*ω*_s_/*ω*_s_, is constant for all the scales. Therefore, at lower frequencies longer wavelets are considered to improve the frequency localization, while at higher frequencies shorter wavelets are used to recover a better time localization. Therefore, the temporal length or the time shift of the wavelet is variable depending on the scale or the frequency range.^57^ However, this implies that since CWT is not completely localized in time, it suffers from edge artifacts.^58^

The number of scales is determined by the number of voices and octaves, where an octave is defined as the frequency range and voices per octave are the number of scales across each octave.^59^ In this study, the number of voices per octave is 40 for all the CWT calculations.

There are numerous kinds of mother wavelets. In this study, we used Generalized Morse Wavelets (GMWs). GMWs are useful in analyzing modulated signals^60^ and are defined by complex analytic wavelet transforms that can preserve information on both amplitude and phase. GMWs use a two-parameter family of wavelets, namely symmetry, *γ*, which determines the wavelet shape and compactness parameter, *β*. GMWs in the frequency domain can be defined as follows (eqn (2)):^61^2
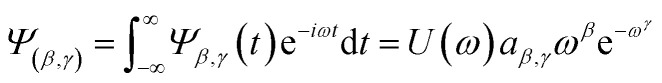
where *a*_*β*,*γ*_ defines a normalization constant and *U*(*ω*) is the unit step function. By changing *β* and *γ*, GMWs are capable of preserving a wide range of signal characterizations while remaining analytic.^62^ Accordingly, one important variable that can be used in defining the behavior of GMWs is the time–bandwidth product which can be calculated using eqn (3).

3 *P*_*β*,*γ*_^2^ = *βγ*

It has been shown that the number of oscillations that can fit into the time-domain wavelet is equal to *P*_*β*,*γ*_/π, where *P*_*β*,*γ*_ represents the wavelet duration or inverse bandwidth. Therefore, defining an appropriate GMW as the mother wavelet depends on the value of *P*_*β*,*γ*_^2^ and *γ*. Different studies have identified that Airy wavelets, GMWs with shape parameter *γ* = 3, have a Gaussian characteristic with high symmetry and concentration of time and frequency.^61,63^ In this study, we used Airy wavelets with *P*_*β*,*γ*_^2^ = 60, where each time-domain wavelet can fit 2.5 oscillations.

Considering a normalized GMW, the magnitude of the complex CWT coefficients is equivalent to the amplitude, eqn (4), and the phase, eqn (5), of the complex value corresponds to the time-related characteristic of the signal, eqn (5).4
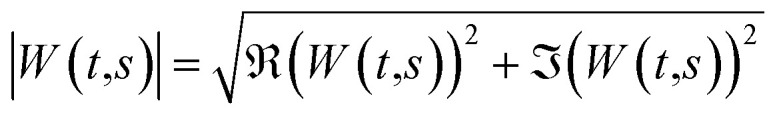
5
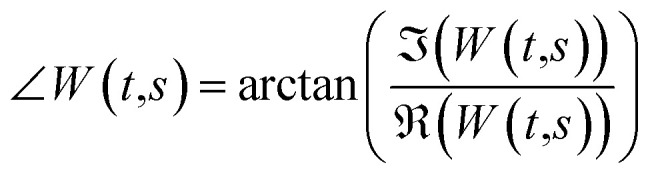
where 
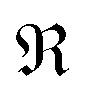
 and 
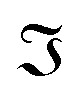
 are the real and the imaginary part of the complex CWT coefficient, respectively. Eqn (5) defines the phase of the complex coefficient; however, in order to find the local phase response of the cantilever, its signal should be compared to the drive signal of the cantilever.

The cross wavelet transform (XWT) calculates the interaction between two time-series signals and is defined as eqn (6).6
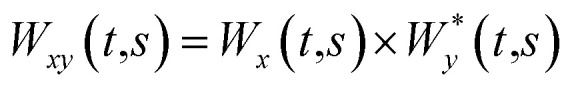
where *x* and *y* are the two time-series signals (*i.e.* cantilever and drive signals), *W* is the CWT coefficient of the signals localized at (*t*,*s*) and W* denotes the complex conjugate of the CWT coefficient.^64^ The *W*_*xy*_(*t*,*s*) argument can define the local relative phase between the cantilever and the drive signal of the cantilever.

A slow varying phase lag can be computed between two signals if they are physically related. Mathematically, wavelet coherence can give information on the local correlation of two time-series.^65^ Therefore, a local phase lag between the two time-series signals using XWT is only valid at the CWT scales where the two daughter wavelets are coherent.

Wavelet coherency determines the coherence of XWT in a time–frequency plane and can be calculated using eqn (7).7
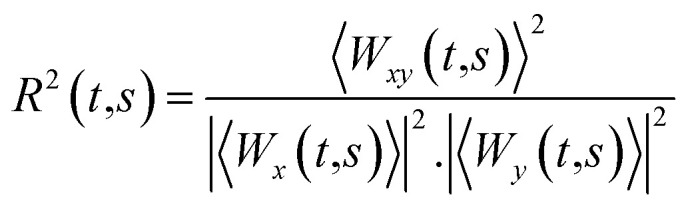
where 〈.〉 denotes the smoothing operator in time and frequency.^65,66^ As shown in eqn (7), wavelet coherence can be regarded as a localized correlation coefficient in the time–frequency plane. Wavelet coherence values are in the range of [0,1], with values closer to one showing a higher correlation between the two signals.

## 3. Materials & methods

### 3.1 Simulation

For simulations, the cantilever-tip motion in dynamic AFM (hence the signal from the photodetector) is described as a driven and damped point-mass oscillator that is under the influence of conservative and non-conservative forces,^40^ and numerical solutions are calculated using a fourth order Runge–Kutta algorithm in C++ software. The equation is described as follows:8
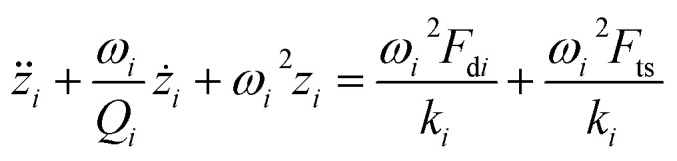
where subscripts *i* = 1, 2 refer to the first and second flexural modes of the cantilever, respectively. *z*_*i*_, *F*_d*i*_, *ω*_*i*_, *Q*_*i*_ and *k*_*i*_ are the tip deflection, drive force, natural frequencies, quality factors and spring constant of the cantilever, respectively. The interaction force is described by conservative and non-conservative viscoelastic force using the following model:^67^9
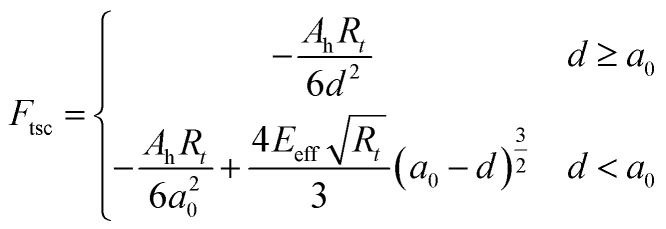
10
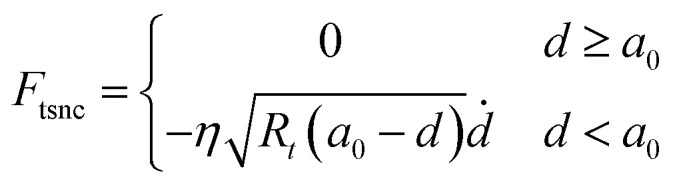
where *A*_h_ is the Hamaker constant, *R*_t_ is the tip radius, *η* is viscosity and 
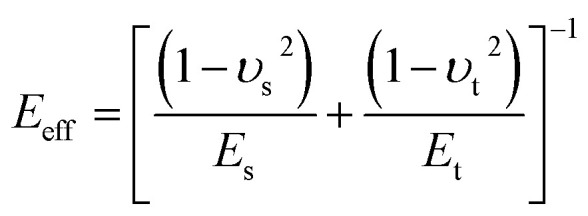
 is the effective Young's modulus of the interaction, where *E*_t_, *E*_s_, *υ*_t_ and *υ*_s_ are the Young's moduli and Poisson's ratio of the tip and sample, respectively. *d* is the minimum distance between the tip and sample and is defined as

11 *d* = *z* + *z*_0_ + *z*_c_

where *z*_c_ is the average distance between the cantilever base and sample surface and *z*_0_ is the average tip deflection. For air environment simulations (considering only the first mode), the free amplitude is 10 nm, frequency is 300 kHz, cantilever spring constant *k* = 30 N m^−1^, quality factor *Q* = 300 and tip radius *R*_t_ = 10 nm. For liquid environment simulations (first and second modes considered, due to the effect of the second eigenmode^68,69^), the free amplitude is 13.5 nm, first and second natural frequencies are 18.6 kHz and 170 kHz, respectively, tip radius (*R*_t_) is 10 nm, spring constants and quality factors of the first and second modes are *k*_1_ = 0.22 N m^−1^ and *k*_2_ = 12.23 N m^−1^, *Q*_1_ = 1.62 and *Q*_2_ = 4.526. While amplitude modulation AFM mapping works by keeping the average probe–sample distance constant by use of a feedback loop, all simulations in the present work are performed assuming no action from the feedback and with varying distance (*i.e.* probe hovering above the surface without any change in absolute *Z* height). In this way topography variations will yield a clear transient signal, which would otherwise depend on the speed of reaction of the feedback.

### 3.2 Experiments

Two samples with distinctly different features have been employed for the present work. The first one (sample 1) is an AFM calibration sample (PNI AFM reference, Pacific Nanotechnology) with patterns of square features with regular size and pitch. The sample is made of silicon nitride film deposited on a silicon substrate. The experiments on this sample were performed on the edge of a 5 × 5 μm^2^ square feature. The second sample (sample 2) is a qualification sample (used to estimate the tip radius of curvature of the employed AFM probes) displaying a granular sharp nanostructure. The sample is made of a hard thin film coating deposited on a silicon chip (TipCheck sample from https://BudgetSensors.com). The experiments were performed in air using a commercial AFM system (D3100 Nanoscope III, Digital Instruments, now Bruker) in amplitude modulation AFM (tapping mode) equipped with a signal access module (SAM III, Digital Instruments) through which the signal from the photodetector could be intercepted. The real-time cantilever vertical deflection signal was acquired using a data acquisition board (NI USB-6366, DAQ Device, National Instruments, Austin, TX, USA). Images of 2 μm scan size with an aspect ratio of 10 (24 × 256 pixels) were acquired at a scan rate of 1 Hz, with a silicon probe for soft tapping mode (FMV-A, Bruker, spring constant 2.8 N m^−1^, resonance frequency 72.96 kHz), at a drive frequency of 72.95 kHz and set amplitude of 85% and recorded at 100 nm lift from the surface. The same parameters were used to acquire a 2 μm image of the reference sample with an aspect ratio of 1 (128 × 128 pixels) at a scan rate of 4 Hz. Deflection sensitivity was calibrated after each set of experiments by recording force curves on the qualification sample.

## 4. Results and discussion

**Fig. 1:**
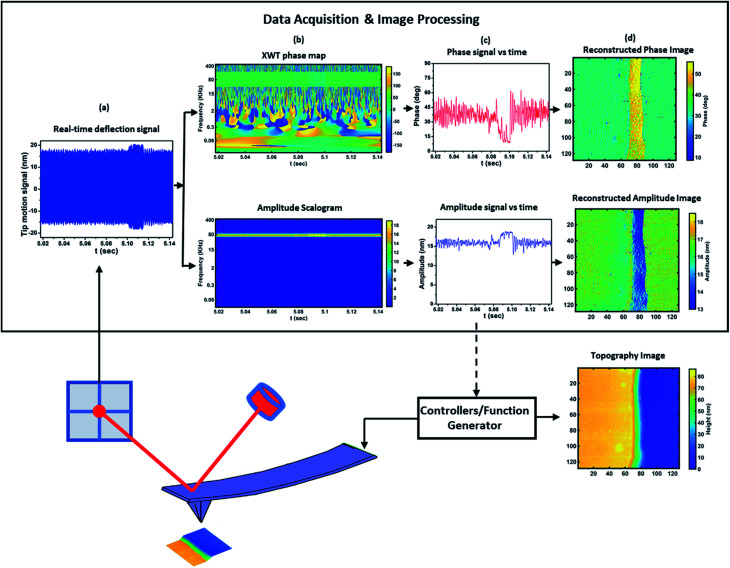
Schematic of the proposed method. Images collected over a single tapping mode scan with the feedback maintaining a set-point amplitude. (a) The real-time signal acquired by the photodetector. (b) Scalogram and time–frequency phase map obtained *via* CWT using the raw signal from the photodetector. (c) Temporal amplitude and phase *versus* time at each frequency extracted from the CWT maps. (d) The amplitude and phase images reconstructed from the temporal signals of the cantilever.

The proposed wavelet transform method, as schematized in Fig. 1, is fed with the cantilever vertical deflection signal as tracked by the AFM photodetector (Fig. 1a). From this signal, the CWT extracts the dynamics of amplitude (magnitude of the CWT coefficients) and phase (XWT) of cantilever motion with respect to time, which can be represented by a scalogram and phase map as shown in Fig. 1b. In this way all the transient and steady state information of the two variables is available. From this collection of information both the transient and averaged amplitude and phase features can be extracted by selecting the data within the corresponding time interval at the frequency of interest (Fig. 1c). Finally, the images of amplitude and phase can be reconstructed from separated signals of phase and amplitude *versus* time (Fig. 1d).

Furthermore, by integrating such method within the AFM system itself, the extracted amplitude information could be used to control the AFM to keep the setpoint value. This would yield an improvement in AFM measurement speed by substituting the lock-in amplifier which has a bandwidth limitation due to the low-pass filter. For this purpose, the CWT can be implemented using FPGA to detect and calculate the phase and amplitude signals in real time from the photodetector signal and provide the amplitude signal as an input of the feedback controller. The integration of implementation of CWT using FPGA^70,71^ and the recent development of FPGA for the control of high-speed AFM^54,72^ represent the applicability of the proposed method for high-speed operation.

**Fig. 2:**
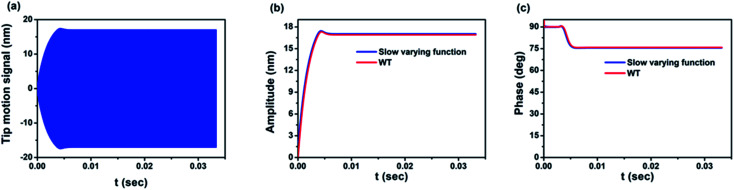
Comparison between simulations obtained using CWT and slow varying function^67^ algorithms: (a) tip motion signal of AFM in the repulsive regime in an air environment, (b) amplitude of the signal calculated by both CWT and slow varying function method^67^ plotted against time, (c) dynamics of the phase calculated by both CWT and slow varying function method^67^ plotted against time.^67^ In this simulation, the AFM parameters are *f*_0_ = 300 kHz, *Q* = 300, *k* = 30 N m^−1^, *R*_t_ = 5 nm, *A*_h_ = 10^−19^ J, *E* = 1 GPa, *a*_0_ = 0.164 nm, *A*_0_ = 20 nm and *z*_c_ = 16 nm.

Firstly, we evaluate the effectiveness and performance of the continuous wavelet transform to provide the amplitude and phase of the signal *versus* time consisting of both transient and steady state regimes. For this purpose, a simulated cantilever deflection signal is converted using CWT and compared with a signal obtained from slow time varying function theory^67^ (Fig. 2), showing very good agreement. It is however worth remarking that the slow time varying method is only applicable for theoretical studies and cannot be applied experimentally, the main advantage of CWT being its ability to be employed in both cases.

**Fig. 3:**
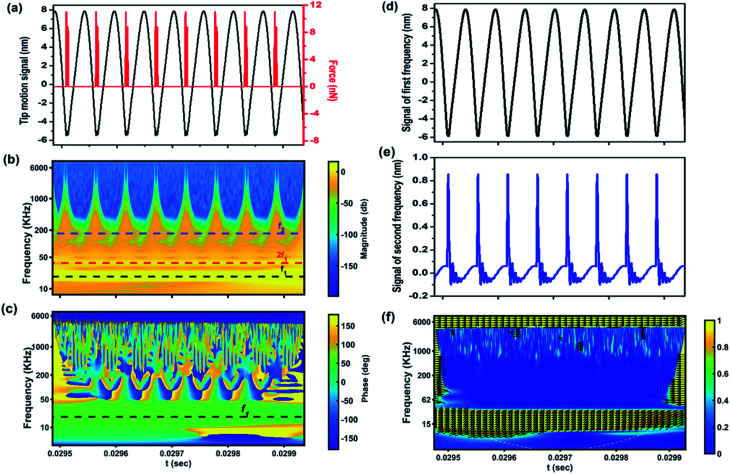
CWT time–frequency analysis of the tip motion signal. (a) The original tip motion and interaction force between the cantilever and sample, (b) the magnitude scalogram of the signal expressed in decibels, (c) the XWT phase map of the simulated signal, (d) signal of the first frequency of the cantilever, (e) signal of the second frequency of the cantilever, and (f) the coherence map. In the scalogram, the first and second eigenfrequencies and second harmonic frequency are represented by the horizontal dashed lines. The simulation parameters are *f*_01_ = 18.6 kHz, *f*_02_ = 170 kHz, *Q*_1_ = 1.62, *Q*_2_ = 4.526, *k*_1_ = 0.22 N m^−1^, *k*_1_ = 12.23 N m^−1^, *R*_t_ = 10 nm, *E* = 10 GPa, and *a*_0_ = 0.164 nm.

The possibility offered by the CWT method to convert a signal into a concise representation is illustrated in Fig. 3. In this case, starting from a simulated signal (Fig. 3a) representing the original cantilever motion, CWT provides information about amplitude and phase into the scalogram (Fig. 3b) and XWT (Fig. 3c) phase maps, respectively. In turn, from the maps the separated signals of the first (Fig. 3d) and second (Fig. 3e) eigenfrequencies of the cantilever are extracted. In this simulation, the effect of the second eigenmode on the original signal is significant and distorts the signal when the cantilever is in contact with the sample, as highlighted in the magnitude scalogram (Fig. 3b). As can be seen from Fig. 3a, during the contact with the sample, the cantilever experiences a repulsive force that excites the second eigenmode of the cantilever. Finally, further calculations yield the wavelet coherence map (Fig. 3f), in which the direction of the arrows show the relative local phase of the cantilever signal in a unit circle, where the coherence is higher than 0.5. Analyzing the XWT and local phase map, in this case, it can be inferred that the phase of the cantilever can be calculated at the first frequency with a wavelet coherence of 1. It is therefore evident from this simple simulation that a distinct advantage offered by employing CWT on an AFM signal is the extraction of information about harmonic effects in the time domain.

**Fig. 4:**
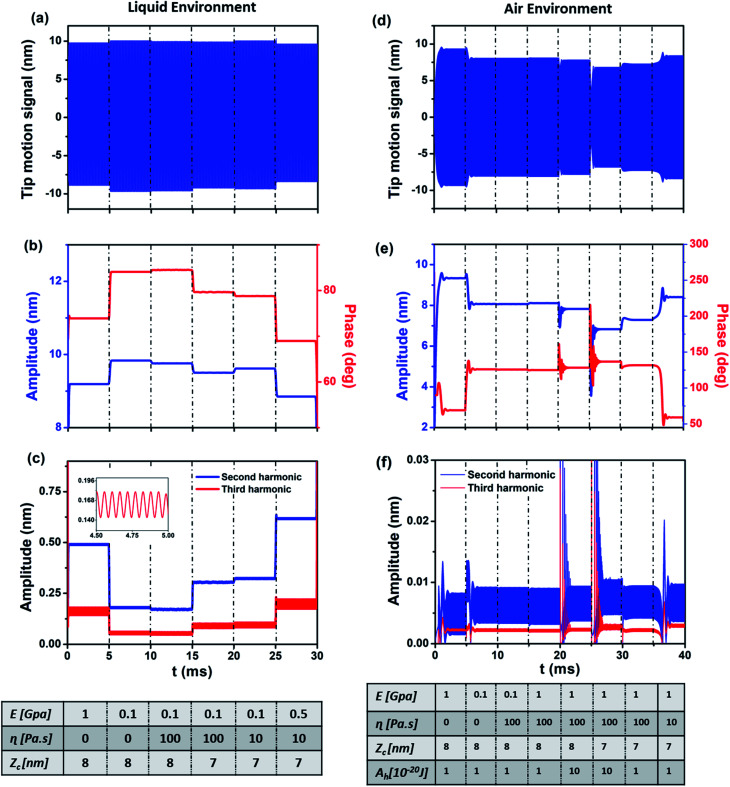
CWT model simulation: comparison between liquid (a, b and c) and air (d, e and f) environments: (a) original signal of tip motion in a liquid environment, (b) amplitude and phase *versus* time response obtained from CWT analysis in a liquid environment, (c) amplitude response of second and third harmonics obtained from CWT analysis, (d) original signal of tip motion in an air environment, (e) amplitude and phase *versus* time response from CWT analysis in an air environment in both attractive and repulsive regimes, (f) amplitude response of second and third harmonics obtained from CWT analysis.

However, the main advantage offered by the CWT is the ability to produce data including information about the transient response of amplitude and phase in relation to the variation of the material's properties and sample topography. To demonstrate it, we performed simulations of the probe–sample interaction both in air and liquid environments (Fig. 4) and applied the CWT method to the resulting signal. The changes across the sample surface have been simulated with varying probe–sample distance *z*_c_ (topography), Young's modulus *E* (stiffness), viscosity *η* and Hamaker constant *A*_h_ (adhesion) at set time intervals.

Fig. 4a–c are the simulated results for the liquid environment and Fig. 4d–f are the simulated results for the air environment. For the simulation in a liquid environment, six different scenarios are considered by changing one of the parameters of the simulation at a time. It starts with average cantilever–sample distance *z*_c_ = 8 nm, free amplitude *A*_0_ = 13.5 nm and the sample's Young's modulus of 1 GPa. After 0.005 seconds, the Young's modulus is set to 100 MPa, leading to an increase in both amplitude and phase. In the case of the phase, a decrease in the material's stiffness means that the repulsive interaction is reduced, giving rise to an increase of phase towards the attractive regime, as we know that in the attractive regime the phase is more than 90°.^67,73,74^ Also, the increase of amplitude is explained by the fact that the probe experiences less force from the sample. In a third step, the viscosity (*η* = 100 Pa s) is included in the simulation. The responses to the change of viscosity are markedly different, with phase slightly increasing and amplitude decreasing. The increase of phase and decrease of amplitude are related to a phase shift due to dissipation in the repulsive regime. At *t* = 0.015 seconds the average distance of cantilever *z*_c_ is decreased, leading to a decrease of both amplitude and phase, meaning that the cantilever experiences slightly more repulsive force. At *t* = 0.020 seconds the viscosity is decreased, which leads to a decrease in phase and increase in amplitude. Finally, the Young's modulus is increased leading to a decrease in both phase and amplitude, meaning more force experienced by the cantilever, *i.e.* increase in repulsive force. From the results it can be summarized that an increase of stiffness or decrease of average probe–sample distance leads to a decrease of both amplitude and phase, while an increase of viscosity leads to an increase of phase and decrease of amplitude. In other words, the behavior of amplitude and phase with respect to change in viscosity is different while for the change in stiffness their behavior is the same. The responses of the amplitude of the second and third harmonics are depicted in Fig. 4c. Both harmonics have the same behavior corresponding to the changes of the simulation parameters while in some cases they have different behaviors with respect to the amplitude of the main frequency, which is interesting to study. As can be seen, decreasing the Young's modulus, in contrast to the main frequency amplitude behavior, leads to a decrease of harmonics which can be expected given that the interaction force is decreased. Increasing the viscosity also slightly reduces the magnitude of harmonics, showing in this case the same behavior for the harmonics and main amplitude. Reducing the average distance leads to an increase of harmonics while the main amplitude is decreased. Then, reduction of viscosity not only increases the main amplitude but also magnifies the harmonics. Finally, increasing the Young's modulus increases the magnitude of harmonics while decreasing the main amplitude. To analyze the behavior of harmonics of the cantilever in a liquid environment due to the changes of parameters, it can be summarized that increasing the Young's modulus and decreasing the average distance – which reduces the main amplitude due to experiencing higher repulsive force – increase the magnitude and effect of harmonics on the main signal. The interesting point of this result is the impact of increase (decrease) of viscosity, or in other words dissipation, on the magnitude of harmonics. With respect to changes in dissipation, the harmonics of the cantilever signal have the same behavior as the main amplitude and increasing (decreasing) the viscosity leads to a decrease (increase) of the amplitude of the second and third harmonics of the cantilever. The effect of the parameter's changes on higher harmonics and second frequency of the cantilever is shown in the ESI.†

To study the behavior of the main amplitude, phase and second/third harmonics of the cantilever in an air environment, eight scenarios are considered. It is worth mentioning that in an air environment, due to the increase of quality factor, the effect on harmonics and eigenfrequencies is very low in comparison with the case of liquid medium. The simulation starts with *z*_c_ = 8 nm, free amplitude *A*_0_ = 10 nm, Hamaker constant = 10^−20^ J and Young's modulus of the sample = 1 GPa.

After 0.005 seconds the Young's modulus of the sample is decreased, resulting in an increase of main amplitude and phase and decrease of second and third harmonics. This is due to the reduction of repulsive force and transition from the repulsive to the attractive regime. An increase of the sample's viscosity to 100 Pa s yields a slight decrease of phase (from 126° to 125.7°) and main amplitude. In contrast to the simulation in a liquid environment, the behaviors of amplitude and phase are the same. This phenomenon has been explained^68,69^ as originating from the difference of phase contrast in air and liquid environments. At *t* = 0.015 seconds an increase of Young's modulus from 100 MPa to 1 GPa leads to an increase in amplitude and decrease in phase. The increase of main amplitude – as is obvious from the phase value – is due to the fact that owing to the interaction in the attractive regime an increase in stiffness leads to a decrease of attractive force, leading to a decrease of phase value below 90° and increase in main amplitude. Increasing the Hamaker constant tenfold, meaning higher attractive force, leads to a slight increase in phase and decrease of main amplitude. At 0.025 seconds a decrease in average distance leads to a decrease in amplitude and increase in phase meaning that the interaction approaches maximum attractive force. Decreasing the Hamaker constant leads to an increase in amplitude and decrease in phase due to the lower value of attractive force. Finally, a decrease in viscosity, moving the interaction into the repulsive regime, increases the amplitude and decreases the phase. In this case the behaviors of phase and amplitude are different, same as in the liquid environment due to the presence of the repulsive regime. The harmonics increase slightly, which means that reduction of dissipation makes the transition from the attractive to the repulsive regime cause different behaviors between phase and amplitude than in the attractive regime.^68,69^ The scalograms of the simulation are given in the ESI.†

**Fig. 5:**
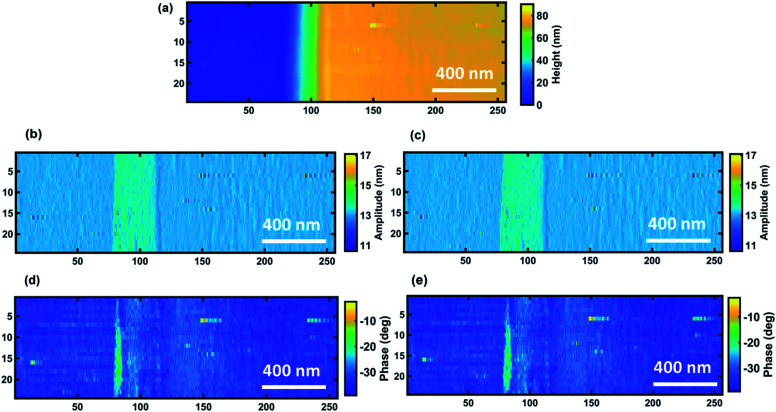
Comparison between standard LIA images (commercial AFM-tapping instrument) and reconstructed ones by CWT for sample 1. (a) The topography of the sample, (b) the amplitude image of the standard LIA, (c) the reconstructed amplitude image calculated by CWT, (d) the phase image of the standard LIA, and (e) the reconstructed phase image calculated by XWT.

After comprehensive analysis of the dynamic response of the cantilever calculated using the continuous wavelet transform on simulated data, we applied the proposed method to the time domain data from actual measurements and compared the results to the data elaborated by the embedded LIA in the AFM system. Amplitude and phase images are generated by CWT as whole signals including transient responses, but for the sake of comparison with the images captured by the AFM system they are reduced to pixel AFM resolution by averaging them over the time interval of each pixel. Performing this operation on one set of data acquired on the calibration sample (sample 1) produces results showing a very good agreement between the two sets of images (Fig. 5), not only for the amplitude but also for the phase signal as well.

**Fig. 6:**
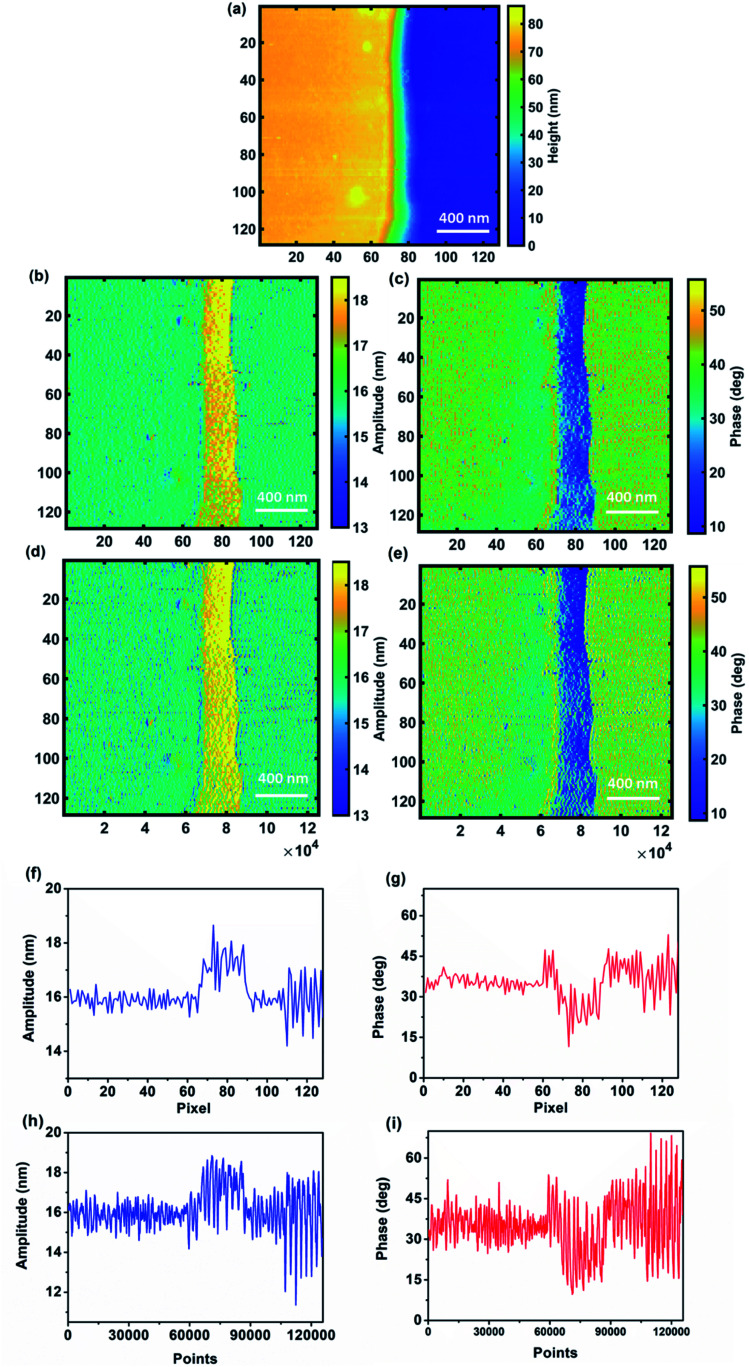
Comparison between the standard averaged image (256 pixel scale – (b), (c), (f) and (g)) and CWT image (whole 120 000 pixel scale – (d), (e), (h) and (i)) for sample 1. (a) topography image of the sample, (b and d) amplitude images and (c and e) phase images. The line scans (f–i) are for line 105 of images.

Non-averaged images obtained by the CWT carry a greater deal of information than the ones provided by the AFM system, mainly due to the fact that the CWT provides a time–frequency analysis of a signal. Therefore, the CWT and its coherence are capable of calculating the amplitude and phase of the signal at all the available datapoints and the only limitation would be the computational cost of applying this signal processing method. Comparison of images obtained by the standard averaging procedure and whole points captured by CWT (Fig. 6b–e), especially between the line profiles of the same line (Fig. 6f–i), not only shows the capability of CWT to provide standard images based on the averaging procedure, but also shows a significant enhancement of the quality and resolution of the temporal CWT images, since the operation of averaging at each pixel leads to information loss in the image with respect to the whole response. Such loss of information becomes critical in case fast dynamic response information from the sample needs to be analyzed. Moreover, computation of the material's properties starting from data extracted from averaged images might be affected and possibly lead to significant error due to the sensitivity of computation at the nanoscale. Note that in this figure we use the same color bar for images based on the maximum and minimum values of averaged images. The same images but considering max and min values of temporal CWT images are shown in the ESI (Fig. S4†) which represents CWT's potential in illustrating the details of an image compared to averaged images. Further details are provided in the ESI section (Fig. S5 and S6†).

**Fig. 7:**
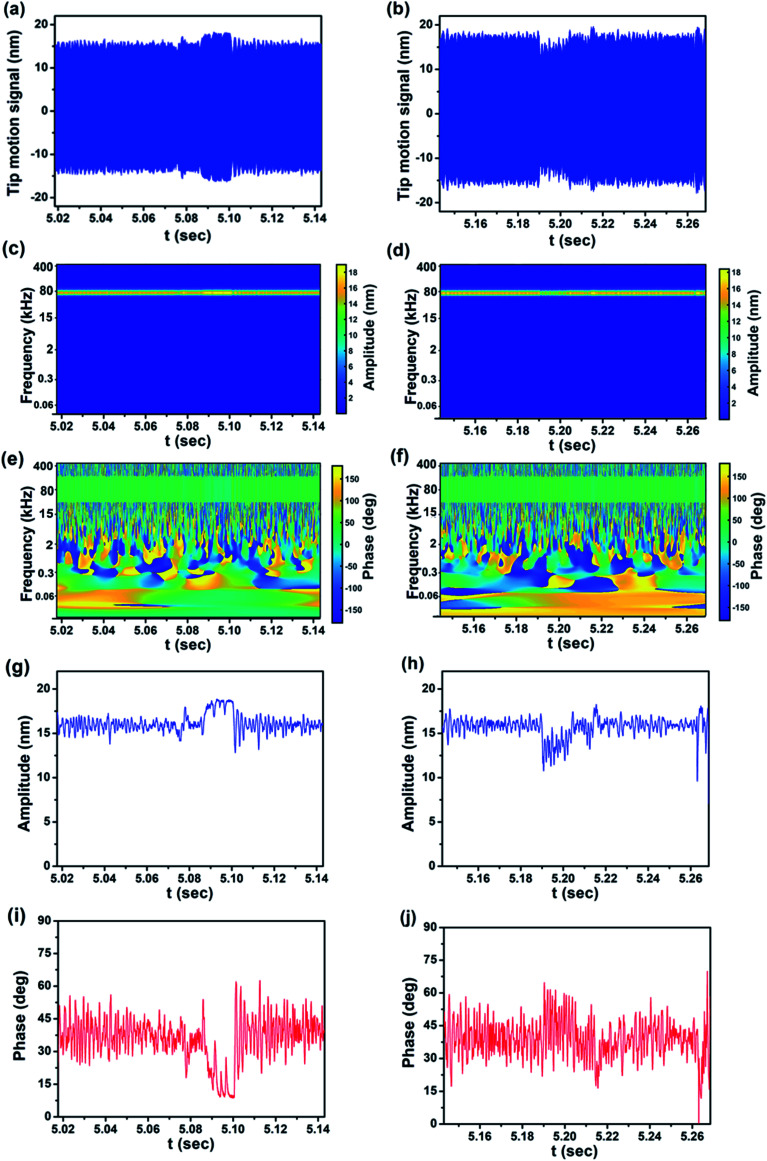
CWT analysis of the experimental signal obtained from imaging sample 1. (a and b) Original cantilever tip motion signal of the trace and retrace, respectively, (c and d) magnitude scalograms of the trace and retrace signals, respectively, (e and f) XWT phase maps of the trace and retrace signals, respectively, (g and h) real-time amplitude response of trace and retrace signals, and (i and j) real-time phase response of trace and retrace signals, respectively.

Fig. 7a and b show the real-time cantilever signal of line 21 of the *Y* axis in the scanned image of Fig. 6 directly obtained using a photodetector. Fig. 7c–f show the information extracted by CWT from the photodetector signal of Fig. 7a and b. Both the trace and retrace of the signal are analyzed, obtaining the magnitude scalogram and XWT phase map. The associated tip motion signals and the transient response of amplitude and phase are given in Fig. 7e–j. It is noteworthy to highlight that with this procedure all the dynamics of the signals of the cantilever are captured and recorded in scalograms and can be recovered at any time. In contrast, with standard AFM data acquisition and processing, the transient response is lost due to averaging and use of stationary spectral analysis. From the behavior of amplitude and phase with respect to time it is completely obvious that the cantilever experiences the height changes of the calibration sample. Comparison between line 21 of the amplitude and phase images with the real-time response of amplitude and phase in Fig. 7g and i shows the effect of the height variation and details of cantilever motion in the response of amplitude and phase which can be easily seen in the results from CWT analysis. It is important to mention that the trace and retrace amplitude and phase signals are direction-dependent, which is easily understood by considering the case of amplitude: the probe passing over an ascending (descending) step will experience a decrease (increase) in amplitude before the feedback adjusts the probe–sample distance. For this reason, at the same step, the amplitude signal is increased in the trace while it is decreased in the retrace direction. For the phase signal the explanation is the same.

**Fig. 8:**
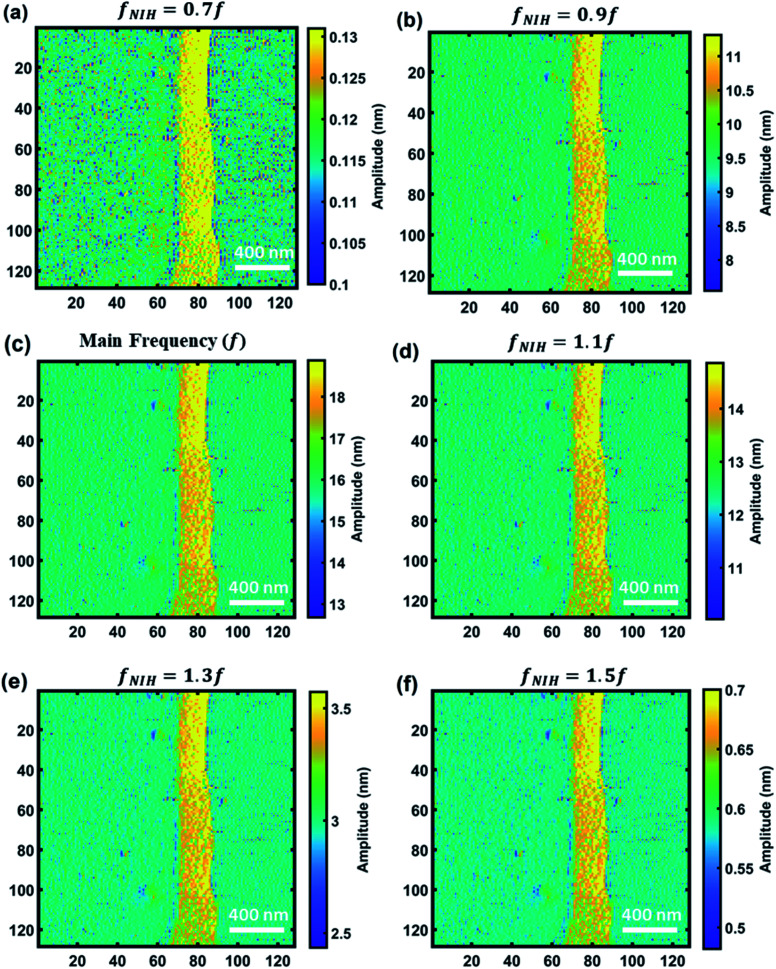
Non-integer harmonic images of sample 1. (a) Amplitude image at 0.7*f* (51 kHz), (b) amplitude image at 0.9*f* (65.6 kHz), (c) amplitude image at the main frequency (*f* = 72.96 kHz), (d) amplitude image at 1.1*f* (80.2 kHz), (e) amplitude image at 1.3*f* (94.8 kHz), and (f) amplitude image at 1.5*f* (109.4 kHz).

In order to show the CWT's capability to provide nonlinear and multifrequency information on cantilever sample interaction, we plot the amplitude maps of non-integer harmonics. Due to the CWT's capability in transforming the signal into daughter wavelets with different scales, where each contains information on a range of frequencies available in the signal, it is possible to plot the images of non-integer harmonics and side band simultaneously in the wide range of frequencies around the excited frequency of the cantilever. The non-integer harmonics are associated with a frequency of transient beats occurring on the cantilever and can provide information about the interaction force and transient instability of the cantilever interacting with the sample surface.^75–79^ The proposed CWT methodology for simultaneously detecting non-integer harmonics, harmonics and eigenfrequencies can provide an opportunity to extract more physical and chemical information from images and acquired data of interaction. Fig. 8 exhibits the non-integer harmonic amplitude images of sample 1 (calibration sample) between 0.7 and 1.5 times the main frequency. As can be seen, the amplitude of non-integer harmonics is different and as the frequency is closer to the main frequency the amplitude increases. For this purpose, the images are plotted in different color scales to better show the data that are saved in different non-integer harmonics. The comparison between non-integer harmonic images shows that the resolution of images in the non-integer harmonics that are higher than the main frequency is better than those with values lower than the main frequency. Moreover, as the non-integer harmonic is closer to the main frequency, the resolution is closer to the image of main frequency. Furthermore, the range of amplitudes in each graph also varies for different non-integer harmonics. A non-integer harmonic of 0.7*f* exhibits the lowest amplitude and lowest range of amplitudes while a non-integer harmonic of 1.1*f* has the closest amplitude and amplitude range to the main frequency. Comparison between 1.3*f* and 0.7*f* shows higher amplitude for 1.3*f* which indicates that for the case of same frequency difference with respect to main frequency, the amplitude of non-integer harmonics with frequency higher than the main frequency is higher than those with lower frequency. From the acquired data, it can be summarized that the distribution and amplitude of non-integer harmonics are not symmetric around the main frequency of the signal which can be related to the nonlinear nature of interaction force. So, CWT can be considered as a versatile tool to study the interplay between a material's properties and nonlinearity and transient response of the cantilever to explore more information about the stability of imaging and extract physical, chemical, and mechanical information from the samples.

**Fig. 9:**
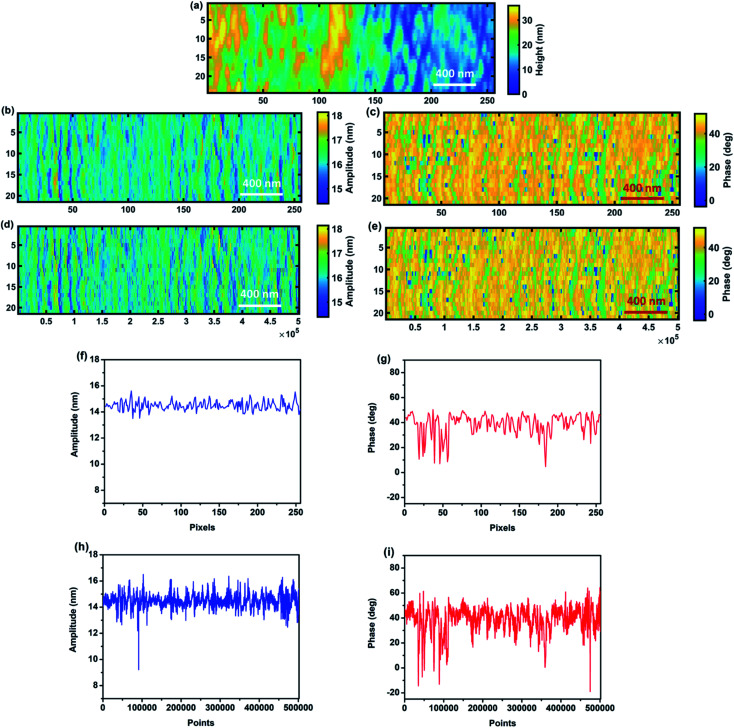
Comparison between standard averaged image (average *x* pixel scale; (b), (c), (f) and (g)) and CWT analysis (whole *x* pixel scale; (d), (e), (h) and (i)) for sample 2. (a) Topography image, (b) averaged amplitude image, (c) averaged phase image, (d) whole amplitude image, and (e) whole phase image of the qualification sample obtained by XWT. (f and g) Averaged amplitude and phase signals and (h and i) real-time amplitude and phase signals of line 14 of the qualification sample captured by CWT.

Finally, in order to show the performance of our method to detect the transient responses in the presence of continuous topography variations, a measurement and analysis on a typical AFM probe qualification sample, sample 2, have been performed. Fig. 9f–i show the averaged and real-time amplitude and phase response of the cantilever of line 14 of the images of Fig. 9b–e. As can be seen here the variation in real-time response of amplitude and phase can be detected precisely, while in the averaged amplitude and phase the data from the signal dynamics are lost. This strongly proves the capability of the proposed technique to detect the transient response of the cantilever. As for the calibration sample measurement and analysis, images and line profile comparison clearly show loss of information during averaging.

**Fig. 10:**
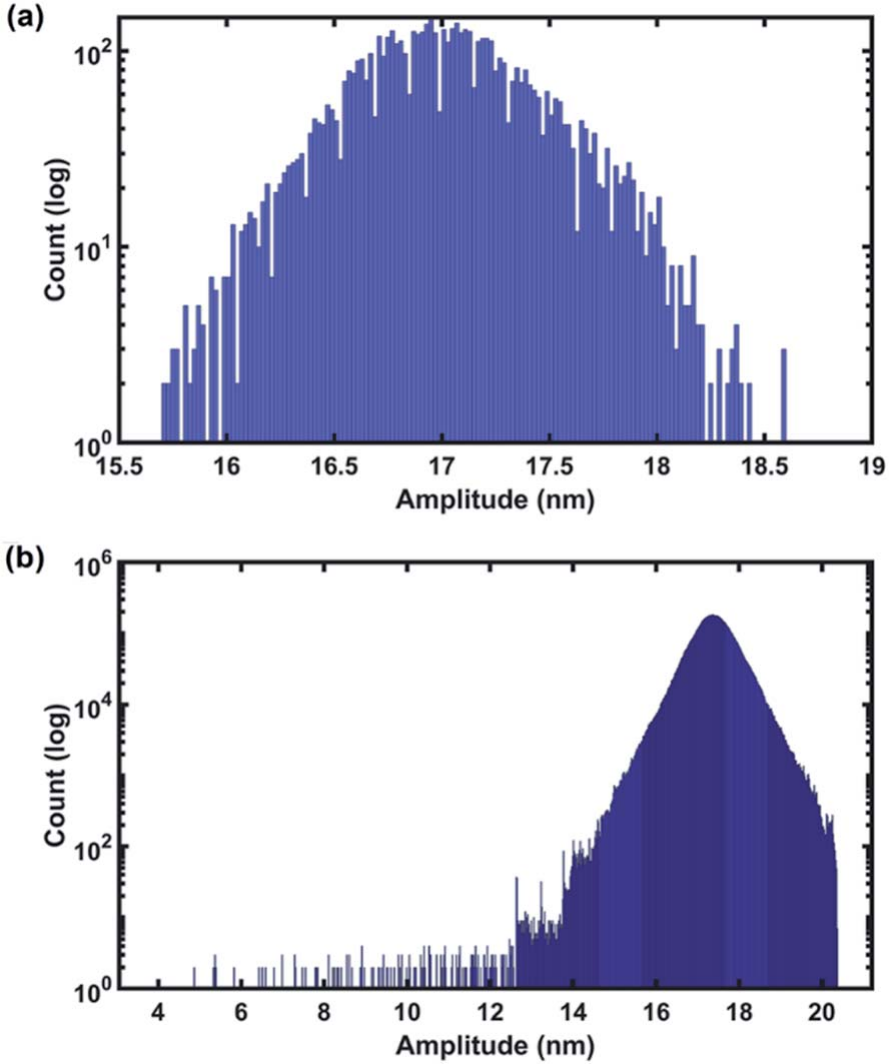
Histogram of amplitudes of the signal detected by (a) the LIA and (b) CWT during imaging.

In order to show the capability of the CWT to detect the transient response of the cantilever we plot the histogram of the amplitudes detected by CWT and compare the data with the detected amplitudes of the standard LIA used in commercial AFMs (Fig. 10). As can be seen, the distribution of amplitudes in the CWT histogram is significantly wider than those of the LIA which means that when using CWT all of the transients and changes that the cantilever experienced can be detected while in the LIA due to its detection mechanism which is based on the steady state regime of the signal and also averaging the data, the transients and fast changes of the signal are lost. Details of the non-integer harmonics of sample 2 are given in the ESI.†

## 5. Conclusion

For the first time the CWT method was used to extract the steady state and transient responses of both the amplitude and phase signals for an AFM cantilever operated in amplitude modulation AFM, providing AFM images informative of the whole dynamics of the tip/surface system.

By use of simulations, we proved the capability of the method to produce data including information about the transient response of amplitude and phase in relation to the variation of a material's properties and sample topography. The technique was successfully used to reconstruct amplitude and phase images of standard samples, starting from time domain data from actual measurements. The results match and surpass in details the images generated by standard LIA analysis.

Finally, this novel concept, by integration within the feedback system of the AFM setup, can be used to control probe movement, opening the path for high-speed transient force microscopy. Substitution of the LIA feedback – with its inherent bandwidth limitation – with the CWT method would firstly improve measurement speed, and secondly reduce information loss, giving access to a wealth of information about transient response, leading to the possibility of analyzing in detail a material's properties in dynamic AFM.

## Conflicts of interest

There are no conflicts to declare.

## Supplementary Material

NA-003-D0NA00531B-s001
