# Supplementary material for: Data acquisition and imaging using wavelet transform: a new path for high speed transient force microscopy
Source: Nanoscale Adv. 2020 Sep 10;3(2):383–98. doi: 10.1039/d0na00531b (PMC9417248; doi:10.1039/d0na00531b)
Supplement: NA-003-D0NA00531B-s001 [file NA-003-D0NA00531B-s001.pdf]

Electronic Supplementary Material (ESI) for Nanoscale Advances.
This journal is © The Royal Society of Chemistry 2020

Supporting Information

Data acquisition and imaging using wavelet transform: a new path for high speed transient force microscopy

Amir Farokh Payam, Pardis Biglarbeigi*, Alessio Morelli, Patrick Lemoine, James McLaughlin, Dewar Finlay*

Nanotechnology and Integrated Bioengineering Centre (NIBEC), School of Engineering, Ulster University, Jordanstown Shore Road, BT37 0QB, Northern Ireland, United Kingdom

Email: a.farokh-payam@ulster.ac.uk

*Equal Contribution

Harmonic analysis of simulation

The scalograms of the simulations in air and liquid environment are given in FigS1. Here, CWT is used in harmonic analysis of dynamic AFM with changes of simulation parameters in liquid and air environments. The scalograms of the CWT, given in Fig.S1, contain information on the amplitude of various frequency ranges available in the signal.

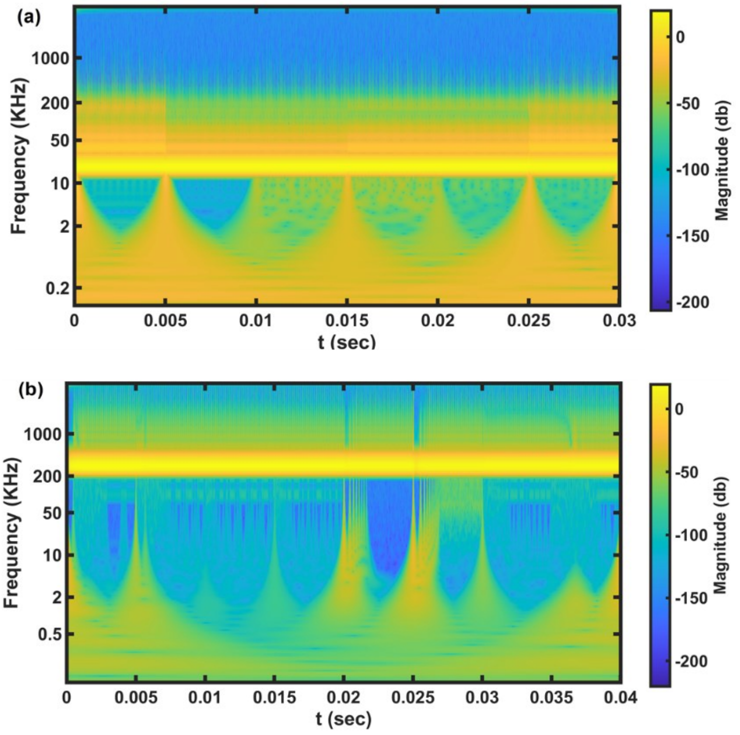

Figure S1. Scalogram of dynamic AFM simulation with changes in parameters a) in liquid and b) in air environments.

From this scalogram the harmonic response of dynamic AFM in liquid is obtained and presented in Fig.S2. As it can be seen, at the second step, decreasing the Young-Modulus leads to the decrease of all the harmonics. Increasing the viscosity at the third step, leads to the slightly decrease of harmonics second to fifth and slightly increase of harmonics sixth to tenth. This increase is related to the effect of second eigenmode and its relation with the phase in liquid which was explained well in (Amir F. Payam, Ramos, & Garcia, 2012). Decrease the average distance at step four leads to the increase of harmonics which is associated to the experience more force. Reduction of viscosity in step five, provides almost the inverse behaviour as step 3. Finally, increasing the stiffness leads to the increase of harmonics. Also, as can be seen from harmonic responses, due to the effect of second eigenmode, the harmonics eighth and ninth are higher than harmonics fifth to seventh and tenth.

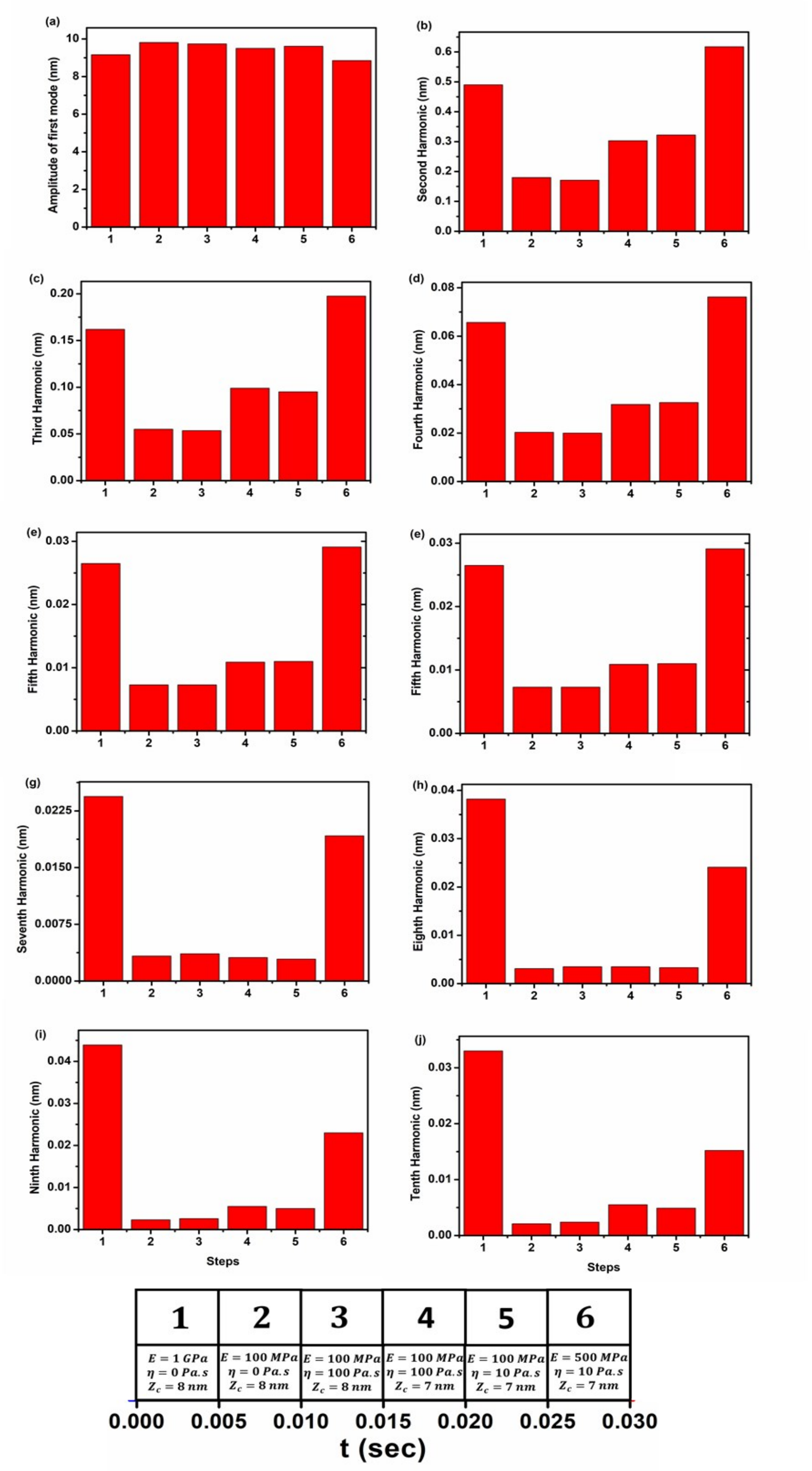

Figure S2. Simulation results of average amplitudes of harmonics. A) Amplitude of first mode, b) second harmonic, c) third harmonic, d) fourth harmonic, e) fifth harmonic, f) sixth harmonic, g) seventh harmonic, h) eighth harmonic, i) ninth harmonic, j) tenth harmonic.

Furthermore, phase response of the simulation can be calculated using cross wavelet transform (XWT), where the simulation has high coherency with the drive signal of cantilever. Fig.S3a represents the coherency map between the two signals. The arrows show the direction of phase lag in a unit circle where the two signals are coherent, i.e. their wavelet coherency is higher than 0.5. Changes in the direction of arrows show the changes in the phase of each simulation step which corresponds to Fig.S3b which the details are given in figure 4 of the paper. This figure shows, in detail, the phase change of each step for the frequency range that the cantilever and drive signal are coherent.

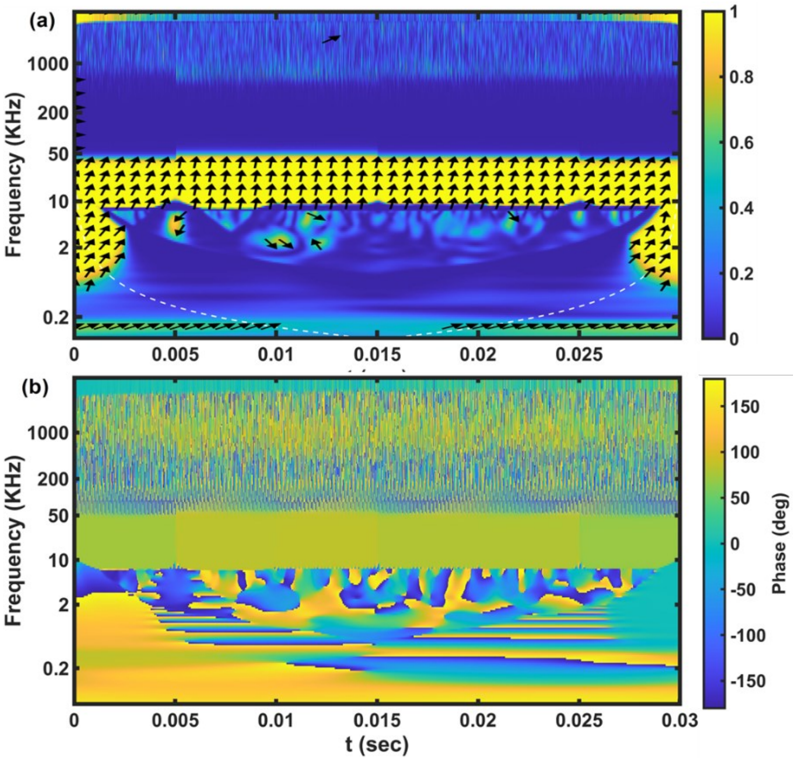

Figure S3. a) coherency map b) phase map obtained by XWT.

Signal analysis of experiments

Figure S4, shows the comparison between averaged images of amplitude and phase and the temporal CWT images. The max and min values for colour bar are chosen based on the temporal CWT images.

As it can be seen, not only temporal CWT images contain the whole points of the captured signal from photodetector, but also their resolution is significantly higher than averaged images.

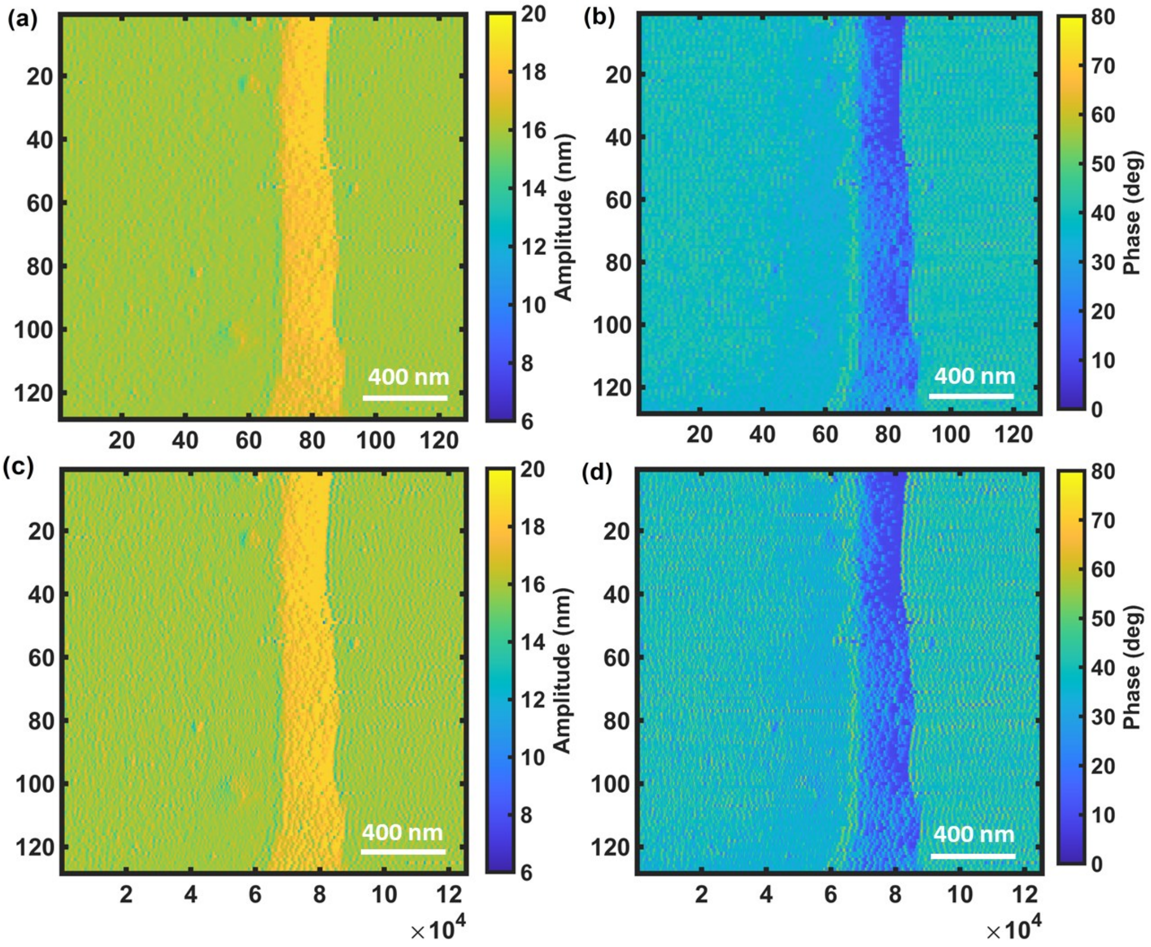

Figure S4. a) and b) Averaged amplitude and phase images, c) and d) temporal CWT amplitude and phase images.

In figures S5 and S6, the real time signals of cantilever-tip motion, its amplitude and phase versus time for both calibration and qualification samples are given.

As it can be depicted, using CWT, the whole information of amplitude and phase is captured and can be used for data processing and acquisition. In this case, all the events that cantilever-tip system sense during the scanning of the surface of the sample are recorded and can be used to explore properties of the sample and produce images. Also, the real time response of harmonics can be detected as shown in figure S6 for second and third harmonics.

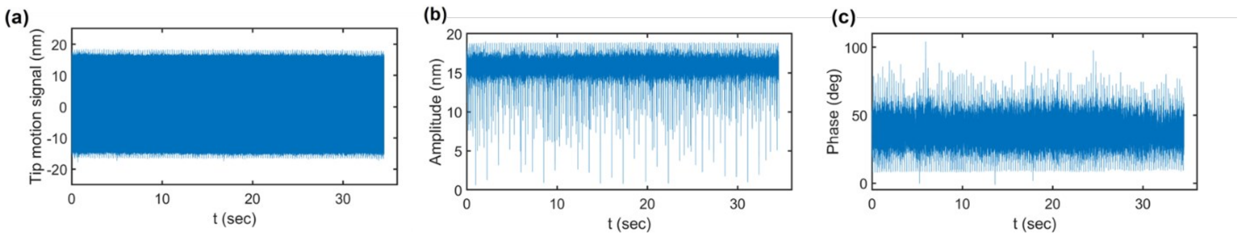

Figure S5. The real time a) cantilever-tip motion signal, b) amplitude and c) phase obtained from CWT of the calibration sample imaging.

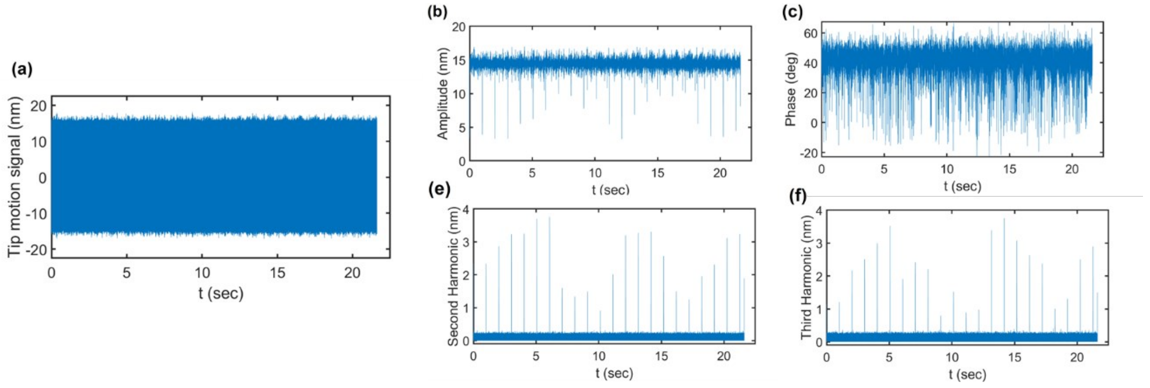

Figure S6. The real time a) cantilever-tip motion signal, b) amplitude, c) phase d) second harmonic, e) third harmonic obtained from CWT of the qualification sample imaging.

The amplitude images of sample 2 captured at different non-integer harmonic frequencies are given in Figure 7S.

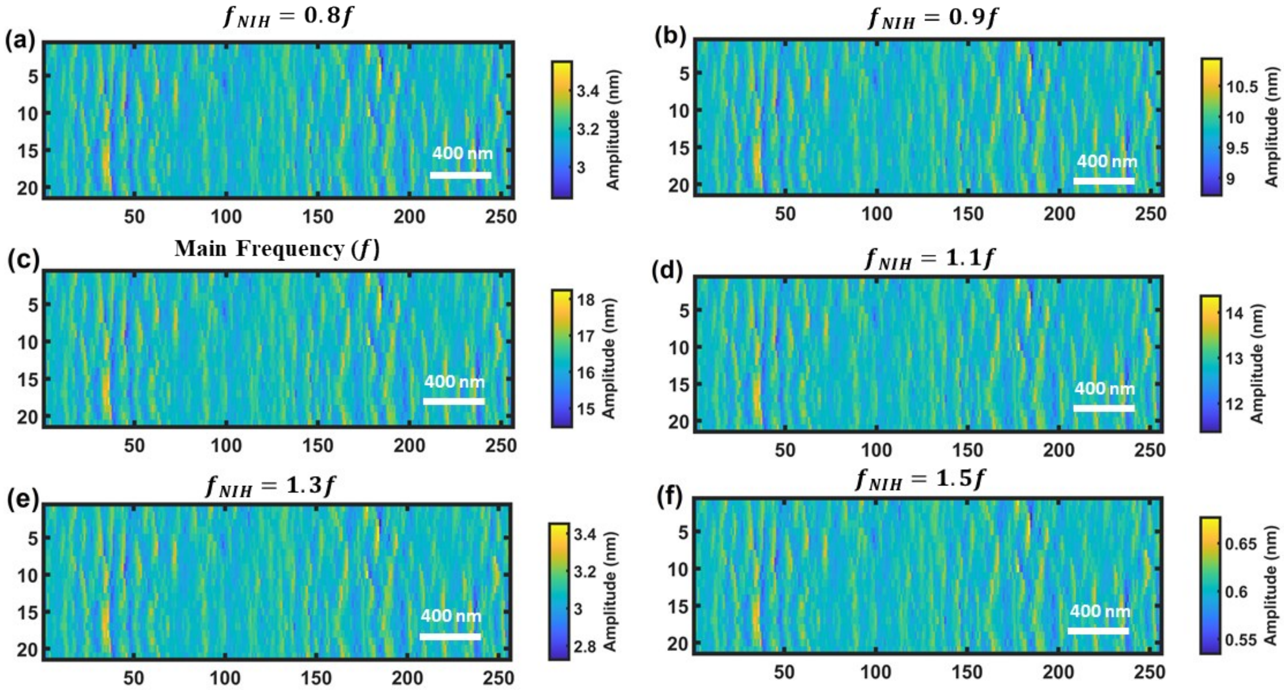

Figure S7. Non-integer harmonic images of sample 2. a) Amplitude image at 0.8f (58.3 kHz), b) amplitude image at 0.9f (65.6 kHz), c) amplitude image at main frequency (f=72.96 kHz) d) amplitude image at 1.1f (80.2 kHz), e) amplitude image at 1.3f (94.8 kHz), f) amplitude image at 1.5f (109.4 kHz).
